# Outcome of Out-of-Hospital Cardiac Arrest Patients Stratified by Pre-Clinical Loading with Aspirin and Heparin: A Retrospective Cohort Analysis

**DOI:** 10.3390/jcm12113817

**Published:** 2023-06-02

**Authors:** Sascha Macherey-Meyer, Sebastian Heyne, Max M. Meertens, Simon Braumann, Stephan F. Niessen, Stephan Baldus, Samuel Lee, Christoph Adler

**Affiliations:** Clinic III for Internal Medicine, Faculty of Medicine and University Hospital Cologne, University of Cologne, 50931 Cologne, Germany; sebastian.heyne@uk-koeln.de (S.H.); max.meertens@uk-koeln.de (M.M.M.); simon.braumann@uk-koeln.de (S.B.); franz.niessen@uk-koeln.de (S.F.N.); stephan.baldus@uk-koeln.de (S.B.); samuel.lee@uk-koeln.de (S.L.); christoph.adler@uk-koeln.de (C.A.)

**Keywords:** OHCA, aspirin, heparin, NSTE-ACS, STEMI

## Abstract

**Background:** Out-of-hospital cardiac arrest (OHCA) has a high prevalence of obstructive coronary artery disease and total coronary occlusion. Consequently, these patients are frequently loaded with antiplatelets and anticoagulants before hospital arrival. However, OHCA patients have multiple non-cardiac causes and high susceptibility for bleeding. In brief, there is a gap in the evidence for loading in OHCA patients. **Objective:** The current analysis stratified the outcome of patients with OHCA according to pre-clinical loading. **Material and Methods:** In a retrospective analysis of an all-comer OHCA registry, patients were stratified by loading with aspirin (ASA) and unfractionated heparin (UFH). Bleeding rate, survival to hospital discharge and favorable neurological outcomes were measured. **Results:** Overall, 272 patients were included, of whom 142 were loaded. Acute coronary syndrome was diagnosed in 103 patients. One-third of STEMIs were not loaded. Conversely, 54% with OHCA from non-ischemic causes were pretreated. Loading was associated with increased survival to hospital discharge (56.3 vs. 40.3%, *p* = 0.008) and a more favorable neurological outcome (80.7 vs. 62.6% *p* = 0.003). Prevalence of bleeding was comparable (26.8 vs. 31.5%, *p* = 0.740). **Conclusions:** Pre-clinical loading did not increase bleeding rates and was associated with favorable survival. Overtreatment of OHCA with non-ischemic origin, but also undertreatment of STEMI-OHCA were documented. Loading without definite diagnosis of sustained ischemia is debatable in the absence of reliable randomized controlled data.

## 1. Introduction

Out-of-hospital cardiac arrest (OHCA) affects 67 to 170 per 100,000 Europeans per year [1,2,3]. It is the third leading cause of death in Europe, and prognosis remains poor despite continual efforts to improve treatment algorithms [1,4]. Only 7–11% of patients in all-comer cohorts survive until hospital discharge, and of these, only few have a favorable neurological outcome [1,2,5,6]. Sudden cardiac death remains the main cause of OHCA and is predominantly driven by atherosclerotic coronary artery disease (CAD) [7]. Recent analyses demonstrated that early coronary angiography in all-comer OHCA cohorts without ST-segment elevation is not superior to a delayed strategy [8,9,10]. However, obstructive CAD is a common finding in OHCA patients, and approximately 20% have acute total coronary artery occlusion [11]. In patients with presumed ongoing ischemia, immediate coronary angiography is still recommended [12,13,14], but identification of ischemia in these comatose patients might be challenging in pre-clinical settings. In cases of return of spontaneous circulation (ROSC) and ST-segment elevation myocardial infarction (STEMI), ischemia detection is feasible with high confidence and diagnostic certainty using electrocardiogram (ECG). In acute coronary syndrome without ST-segment elevation (NSTE-ACS), specific clinical criteria are missing. Chest pain is a suggestive symptom, but is non-specific with multiple non-cardiac causes [11,15,16]. Pre-hospital measurement of troponin is technically feasible but not routinely established yet [17,18]. Consequently, pre-clinical suspected diagnosis of NSTE-ACS is often false positive, and patients are at risk for overtreatment [19,20]. In Germany, patients with suspected NSTE-ACS are frequently pretreated by pre-hospital application of aspirin (ASA) and/or unfractionated heparin (UFH)—so called “loading” [19,20,21]. There is a gap in the evidence for loading in OHCA patients with high prevalence of obstructive CAD, but also other potential causes of cardiac arrest, with high susceptibility for bleeding.

The current analysis aimed to stratify the outcome of patients with OHCA from a single cardiac arrest center according to pre-clinical loading decision.

## 2. Material and Methods

This retrospective, single-center study is based on a registry of consecutive OHCA patients treated at our cardiac arrest center located in the department of cardiology in a tertiary hospital. The registry generally included all-cause OHCA patients treated at our cardiac arrest center between January 2014 and November 2021, and the vast majority was treated at the department of cardiology. Of these, the majority suffer from OHCA of cardiac origin, as emergency medical services frequently allocate these patients to our hospital to provide extracorporeal cardiopulmonary resuscitation (eCPR). Patients with pre-hospital ROSC, but also under ongoing manual or mechanical CPR, were considered. Adult patients with non-traumatic cardiac arrest and complete information on pre-clinical loading and in-hospital course were eligible for this analysis.

### 2.1. Treatment Algorithm

Our cardiac arrest center is located in a metropolitan area with approximately 1.1 million inhabitants. The contributing emergency medical service (EMS) covers a 400 km^2^ area. In case of pre-hospital cardiac arrest, EMS personnel will be supported by a specialized and trained German emergency physician (EP) leading resuscitation. During ongoing resuscitation, the EP decides whether to stay on scene and continuing pre-clinical treatment until ROSC or termination of cardiopulmonary resuscitation (CPR), or to transport the patient using mechanical CPR (mCPR) devices. According to our local protocol [22], patients with non-traumatic OHCA from presumed cardiac origin are immediately transferred to the catheterization laboratory. Adjudication of cardiac origin is at the discretion of the treating EP after consultation of a cardiologist by phone. In patients with ROSC, the need for urgent coronary angiography is evaluated versus direct transfer to an intensive care unit (ICU). In refractory cardiac arrest or intermittent ROSC, patients will be evaluated by an interdisciplinary heart team for implementation of eCPR or mechanical circulatory support (MCS). Patients with non-cardiac OHCA (e.g., hypothermia, drowning, intoxication) are transferred to the emergency department for further evaluation and are subsequently treated at the ICU. Loading with ASA and UFH, and dosage were at treating EP’s discretion.

### 2.2. Measured Data and Investigated Outcomes

Baseline characteristics were extracted from patient records, including age and gender, data on pre-emergency status, detailed information on resuscitation and pre-hospital treatment, in-hospital outcome data and reasons for cardiac arrest. Arterial blood gas measurements and partial thromboplastin time (PTT) were also extracted. Patients were then stratified according to pre-clinical treatment with ASA and UFH. Additionally, subgroup analysis of STEMI patients was performed, as STEMI guidelines recommend immediate use of antithrombotics and anticoagulants at the time of diagnosis even in pre-clinical settings [13]. Measured outcomes were survival at hospital discharge and favorable neurological outcome at hospital discharge (defined by the Glasgow–Pittsburgh cerebral performance categories (CPC) Score ≤ 2), bleeding complications (defined as a composite of need for red blood cell [RBC] transfusion and intracranial bleeding), ICU and hospital stay. Ethical approval was not necessary in this retrospective, non-interventional analysis of the local OHCA registry.

### 2.3. Statistical Analysis

Data were described using mean values (±standard deviation), or frequencies and percentages. Student’s *t*-test, Fisher’s exact test and chi-squared test were used for statistical analyses according to metric or categorial variables. All reported *p*-values were two-sided, and *p*-values less than 0.05 were considered statistically significant. Statistical analyses were performed using SPSS Statistics Version 27.0.0 (IBM Corp., Armonk, NY, USA).

## 3. Results

### 3.1. Overall Analysis

**Figure 1:**
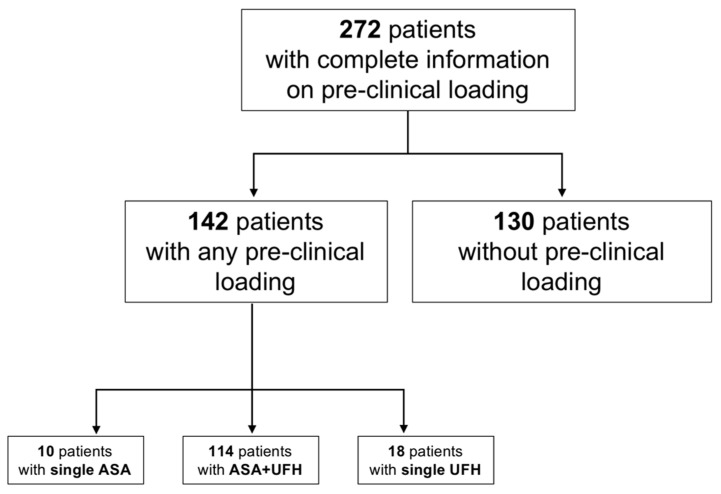
Patient cohort. Abbreviations: ASA: aspirin, UFH: unfractionated heparin.

Overall, 272 patients were included in the registry analysis (Figure 1). Patients had a mean age of 62.7 years and were more frequently male (*n* = 196) (Table 1). Cardiac arrest was witnessed in 211 (77.6%) patients, and 170 (62.5%) received prompt bystander CPR. Shockable rhythm was present in 174 (64%) patients, and they required a mean of 3 shocks. ROSC could be achieved in 245 (90%) patients, and mean time until ROSC was 26.7 min. mCPR was implemented on-scene or during transport in 75 (27.6%) patients. Immediate coronary angiography was performed in 229 (84.2%) patients, and 48 (17.6%) patients required MCS.

Cardiac etiology was the main reason for OHCA. In detail, 103 (37.9%)) patients were classified as having acute coronary syndrome (ACS), 60 presented with STEMI and 43 with NSTE-ACS. Arrhythmia (*n* = 76, 27.9%) was the second leading cause of OHCA, followed by asphyxia (*n* = 28, 10.3%). The remaining 65 (23.9%) patients represented a heterogeneous group mainly suffering from distributive, hypovolemic or obstructive shock.

### 3.2. Loading Status

ASA and/or UFH was used in 142 patients, and 130 did not receive loading before hospital admission (Figure 1). Dosage of aspirin varied between 125 mg and 725 mg. UFH was administered at 5000 or 10,000 units, except in one case in which the EP used 24,000 units.

Patients in the loading group more often had witnessed arrest, were more often treated with bystander CPR, more frequently had ACS and subsequently had a higher proportion of coronary angiography (Table 1). Amiodarone use was more often documented in patients without pre-hospital loading. All other characteristics were distributed evenly between the groups.

Safety analysis showed comparable incidence of bleeding events (26.8 vs. 31.5%, *p* = 0.740) between the groups. This event rate was mainly driven by RBC transfusion (25.4 vs. 28.5%, Table 2). Intracranial bleeding was detected in 2.8% and 5.4% patients by computed tomography (*p* = 0.553).

Both groups had comparable duration of ICU stay, but patients in the loading group showed a trend towards longer overall hospital stay (14.0 vs. 11.1 days, *p* = 0.07).

Patients in the loading group had a significantly higher rate of survival to hospital discharge (56.3 vs. 40.3%, *p* = 0.008). They additionally had a more favorable neurological outcome (80.7 vs. 62.6% *p* = 0.003) compared to patients without loading.

### 3.3. STEMI Subgroup Analysis

Grouping of STEMI patients according to loading status resulted in 40 patients with pre-clinical loading and 20 patients without pretreatment. Patients in the loading group had numerically lower incidence of bleeding events (25 vs. 55%, *p* = 0.212), and especially decreased need for RBC transfusion (Table 3). These observations showed no statistically significant differences. Rate of survival to hospital discharge (77.5% vs. 60%) or favorable neurological outcome (94.1% vs. 82.4%) and hospital or ICU stay were more favorable in the loading group, but were not statistically different.

## 4. Discussion

To our knowledge, this is the first systematic evaluation of pre-hospital loading with aspirin and heparin in OHCA. These are the main and novel findings:

In highly selected patients with mainly cardiac origin,

▪ loading was associated with increased survival to hospital discharge and a more favorable neurological outcome,
▪ the rates for RBC transfusion and intracranial bleeding were not affected by pre-clinical loading,
▪ a considerable number of STEMI patients (33%) were not loaded on scene,
▪ 54% of patients in the loading group had OHCA from non-ischemic cause and had no expected benefit from pretreatment, retrospectively.

Patients in the loading group had an advantageous survival and neurological outcome. Given the non-randomized, uncontrolled registry design, these observations need to be interpreted with caution. The study group represents a highly selected cohort. Rates of witnessed cardiac arrest, bystander CPR and shockable rhythm were high compared to an all-comer cohort [1,3,23]. The majority of patients were male. EMS was activated and CPR was started in each patient. All patients were transported to a hospital, and ROSC could be achieved in a considerable number of patients. Moreover, patients were young and the cohort had a high prevalence of ACS. These are all well-known favorable prognostic factors in OHCA [1,5,6,7,24], and might have contributed to observed favorable survival and neurological outcome rates in both groups. The groups were not balanced in these important characteristics. Hence, advantageous outcome of the loading group is attributable to the increased rate of witnessed arrest, high percentage of bystander CPR and higher prevalence of ACS than to loading itself.

### 4.1. Bleeding

Bleeding rates were similar between the groups. Notably, the registry design underestimates the true prevalence of bleeding complications by assessing only RBC transfusion and intracranial bleeding. In the literature, intracranial bleeding has a prevalence of 3.5 to 11.5% in OHCA patients [25,26,27], and intracranial hemorrhage itself can be the cause of OHCA [25,28]. Overall bleeding complications in OHCA range from 15–20%, and increase to 31–32% in patients treated with eCPR or MCS [29,30,31,32]. Mechanical CPR and MCS themselves are associated with increased bleeding risk, and in MCS, access-site bleeding is a frequent complication [33,34]. Current registry data are in line with prior publications, but one should bear in mind that RBC transfusion is an unspecific bleeding event and OHCA patients are at increased bleeding risk even in the absence of antithrombotic/anticoagulatory pretreatment. Future evaluation of loading harm should ideally address all entities of bleeding.

### 4.2. Undertreatment of STEMI

One-third of STEMI patients were not treated with ASA or UFH in the current registry, even though current guidelines recommend immediate loading at the time of diagnosis [13]. In STEMI patients—in whom coronary artery occlusion is likely—pre-hospital administration of heparin did not affect clinical outcomes in prior analyses. Heparin use led to fewer coronary artery occlusions, but major adverse cardiac events or 30 day survival were not affected [35,36,37]. In accordance, the current analysis did not reveal clinically significant differences between loaded and non-pretreated STEMI group. At the patient level, the reasoning for withholding ASA and UFH in STEMI remains unclear, but some factors could be involved. First, simple misdiagnosis of ST-segment elevation in pre-hospital settings is a possible explanation. Notably, even extracardiac pathologies like intracranial hemorrhage can mimic transient ischemic ECG patterns like ST-segment elevation and might be misleading [38]. Misjudgment of STEMI equivalents (e.g., posterior infarction) or misinterpretation of bundle branch blockade or paced rhythms are also potential factors. Electrolyte imbalances, conduction disturbances or use of antiarrhythmic drugs might have contributed to bizarre ECG presentations. Of note, Baldi et al. demonstrated that immediate ECG following ROSC can be misleading, showing both false negative or false positive STEMI results [39]. Consequently, these authors recommend delayed ECG acquisition for eight minutes following ROSC to minimize systematic diagnostic errors [39].

### 4.3. Overtreatment of Non-STEMI OHCA

More than 50% of patients in the loading group had a non-ischemic cause of OHCA. One might assume that loading was not beneficial in these patients, even though the current study was not designed to demonstrate such difference.

In subjects with preclinically unknown intracavitary bleeding or aortic dissection, the administration of antiplatelets or anticoagulants might cause severe harm. Patient selection for pre-treatment is of utmost interest. Current guidelines on NSTE-ACS or resuscitation do not explicitly address pre-hospital loading. To date, only position papers are available. A position paper from the Acute Cardiovascular Care Association of the European Society of Cardiology recommends pre-hospital loading with aspirin and heparin in STEMI and NSTE-ACS with immediate invasive strategy (coronary angiography < 2 h) [40]. The authors point out that there is no scientific evidence for pre-hospital loading in NSTE-ACS patients, overall. Specific recommendations for OHCA are also missing.

Given this vacuum, STEMI and NSTE-ACS guidelines should be considered in OHCA with presumed cardiac cause [13,14]. The European guideline on NSTE-ACS recommends the use of aspirin, and the administration of UFH at the time of diagnosis [14,41]. Troponin measurement is a cornerstone in NSTE-ACS diagnosis [14], but it is not routinely used on scene in daily practice [17,18]. As a consequence, NSTE-ACS remains solely a suspected diagnosis in OHCA patients on scene, but this judgment affects upcoming treatment steps in the chain of survival. The paradigm shift from immediate to delayed coronary angiography in hemodynamic stable NSTE-ACS-OHCA patients currently translates to daily routine [8,9,10]. Identification and discrimination of patients with total coronary artery occlusion is challenging but crucial. One might speculate that these patients still require immediate coronary angiography including percutaneous coronary intervention. Guidelines recommend immediate angiography in patients with infarct-related cardiogenic shock with hemodynamic instability, ongoing chest pain, life threatening arrhythmias or mechanical infarct complication, but diagnostic modalities are limited in pre-hospital settings [14]. EPs need to assess the individuals’ probability of ischemia on clinical criteria. Spirito et al. recently showed that hemodynamic instability does not automatically indicate vessel occlusion [11]. Instead, shockable rhythm and presence of chest pain demonstrated a predictive value for coronary artery occlusion [11]. However, chest pain is not reliably assessable in comatose patients, especially in patients with non-witnessed collapse. In all-comer chest pain cohorts, non-ischemic and even non-cardiac are the most prevalent causes [15,16]. The retrospective data from Spirito and colleagues must be weighed against this non-specific character of chest pain. Diagnostic error remains the Achilles heel of optimized OHCA patient management, and medical history and evidence of chest pain might contribute to decision making, but prospectively validated criteria are missing.

We observed heterogeneity in loading of OHCA patients with both under- and overtreatment. Nescience and uncertainty of EPs are potential mechanisms. In the absence of reliable randomized controlled data, use of pre-hospital antithrombotic and anticoagulatory pretreatment without ischemic cardiac cause of OHCA remains debatable. Future studies should address clinically measurable factors to overcome these gaps in the evidence. These could possibly change the current strategy from unselected to individualized, selected loading strategies in OHCA patients with considerable risk for coronary artery occlusion.

### 4.4. Limitations

The current analysis followed a non-controlled design in a relatively small cohort. Hence, selection and performance bias are inherent limitations and restrict generalizability. As previously mentioned, the current cohort had a high prevalence of favorable prognostic factors and the majority of patients suffered from ACS. The performance bias was mainly based on loading decision. Administration of ASA and UFH was solely at the treating EP’s discretion. Especially in STEMI patients without loading, individual reasoning remains uncertain, but might reflect uncertainty of the EP. As our registry does not regularly include all entities of bleeding events, we decided to only report the routinely measured data (RBC transfusion, intracranial hemorrhage). In doing so, we numerically underestimated bleeding rates of missing events like gastrointestinal, parenchymatous, intrathoracic, abdominal or access-site bleeding. The relatively small sample size restricts statistical power.

## 5. Conclusions

In this non-traumatic OHCA registry including mainly patients with cardiac cause pre-clinical loading was neither associated with increased intracranial bleeding, nor resulted in higher requirement of red blood cell transfusion. Overtreatment with aspirin and heparin could be documented in 54% of patients presenting with OHCA of non-ischemic origin. One-third of STEMI-OHCA were not loaded, but this undertreatment did not translate to worse survival. The administration of anticoagulatory and antithrombotic pretreatment in OHCA without definite diagnosis of sustained ischemia is debatable in the absence of reliable randomized controlled data. Future prospective studies should address the loading dilemma and evaluate benefit and harm of pre-hospital loading in comatose patients.

## Figures and Tables

**Table 1:** Patient characteristics total cohort.

	Total Cohort N = 272	Pre-Clinical Loading N = 142	No Loading N = 130	*p*-Value *
Age, mean [SD]	62.7 [±15.5]	61.5 [±14.9]	64.1 [±16.4]	0.168
Gender male (%)	196 (72.1)	108 (76)	88 (67.7)	0.125
Pre-emergency status (%)				0.255
*No prior diseases*	50 (18.4)	31 (21.8)	19 (14.6)	
*Diseases without limitations in daily living*	134 (49.3)	72 (50.7)	62 (47.7)	
*Diseases with limitation in daily living*	41 (15.1)	17 (12)	24 (18.5)	
*No independent daily living*	4 (1.5)	1 (0.7)	3 (2.3)	
*Unknown status*	43 (15.8)	21 (14.8)	22 (16.9)	
**Pre-hospital characteristics**				
Witnessed arrest	211	116	95	**0.037**
Bystander CPR	170 (62.5)	97 (68.3)	73 (56.2)	**0.039**
No-flow time, min	2.2 [±4]	2.1 [±4.1]	2.4 [±4]	0.624
Shockable rhythm	174	99	75	0.114
Shocks, n	2.99 [±3.5]	3.1 [±3.7]	2.9 [±3.3]	0.649
Epinephrine use, n	214	109	105	0.460
Amiodarone use, n	108	49	59	**0.008**
Achieving ROSC before hospital arrival ^§^	190	100	90	0.914
Achieving ROSC after hospital arrival ^§^	55	28	27	
Never ROSC achieved ^§^	25	14	11	
Time until ROSC, min	26.7 [±22.9]	26.4 [±25.5]	27.2 [±19.8]	0.795
EMS transport with mechanical cardiopulmonary resuscitation device	75	40	35	0.854
**Presenting arterial blood gases, means**				
Initial arterial O_2_, mm Hg	182.5 [±106.5]	176 [±88.3]	190.4 [±125.7]	0.419
Initial arterial CO_2_, mm Hg	59.8 [±27.9]	58.7 [±25.2]	60.6 [±88.3]	0.829
Initial lactate, mmol/L	7.98 [±6]	7.36 [±5.9]	8.67 [±6.0]	0.073
Initial pH	7.15 [±0.2]	7.17 [±0.2]	7.12 [±0.2]	0.166
Initial hemoglobin g/dL	14.3 [±2.9]	15.1 [±2.3]	13.5 [±3.3]	0.135
**In-hospital treatment**				
Coronary angiography performed (%)	229 (84.2)	129 (90.8)	100 (76.9)	**0.002**
Mechanical circulatory support implantation (%)				
*ECMO*	33 (12.1)	16 (11.3)	17 (13.1)	0.631
*Axial flow pump (Impella©)*	8 (2.9)	6 (4.2)	2 (1.5)	0.286
*IABP*	7 (2.6)	4 (2.8)	3 (2.3)	1.000
Target temperature management	89 (32.7)	48 (33.8)	41 (31.5)	0.691
PTT, s	50.2 [±31.7]	57 [±31.4]	45.1 [±31.1]	**0.014**
Aspiration pneumonia, n	110	55	55	0.565
Hypoxic ischemic encephalopathy (%)	64 (23.5)	24 (16.9)	40 (30.8)	**0.007**
Ejection fraction, EF (%)				0.101
*Preserved EF (≥50%)*	83 (30.5)	42 (29.6)	41 (31.5)	
*Mildly reduced EF (41 to 49%)*	42 (15.4)	27 (19)	15 (11.5)	
*Reduced EF (≤40%)*	86 (31.7)	48 (33.8)	38 (29.2)	
*Not estimated*	61 (22.5)	25 (17.6)	36 (27.7)	
**Cause of non-traumatic cardiac arrest (%)**				**0.019**
*Acute coronary syndrome* - *STEMI* - *NSTE-ACS*	103 (37.9) 60 43	65 (45.8) ^#^ 40 25	38 (29.2) ^#^ 20 18	0.005 ^#^
*Arrhythmia*	76 (27.9)	32 (22.5)	44 (33.8)	
*Asphyxia*	28 (10.3)	11 (7.7)	17 (13.1)	
*Other*	65 (23.9)	34 (23.9)	31 (23.9)	

* Chi-square test/Fisher’s exact test in categorical variables and *t*-test in metric variables. [] Standard deviation, () Percentages. ^#^ ACS vs. non-ACS. ^§^ missing Data: n = 2. Abbreviations: ACS: acute coronary syndrome; CPR: cardiopulmonary resuscitation; ECMO: extracorporeal membrane oxygenation; EF: ejection fraction; EMS: emergency medical service; IABP: intra-aortic balloon pump; NSTE-ACS: non-ST-segment elevation acute coronary syndrome; PTT: partial thromboplastin time; ROSC: return of spontaneous circulation, STEMI: ST-segment elevation myocardial infarction.

**Table 2:** Outcome of patients stratified by loading.

	**Pre-Clinical Loading** **N = 142**	**No Loading** **N = 130**	*p*-Value *
Patients with bleeding complication, n (%)	38 (26.8)	41 (31.5)	0.740
*RBC transfusion, n patients (%)* *Mean number of RBC transfusion*	36 (25.4) 2.4 [±7]	37 (28.5) 2.7 [±7]	0.587 0.722
*Intracranial bleeding, n*	4	7	0.553
ICU stay, mean	6.3 [±5.5]	7 [±5.8]	0.557
Hospital stay, mean	14 [±14.5]	11.1 [±10.8]	0.07
Survival to hospital discharge, % total group	56.3	40.3	**0.008**
Favorable neurological outcome at hospital discharge, % of survivors	80.7	62.6	**0.003**

Abbreviations: RBC: red blood cell; ICU: intensive care unit. * Chi-square test/Fisher’s exact test in categorical variables and *t*-test in metric variables.

**Table 3:** Outcome of STEMI patients stratified by loading.

	Pre-Clinical Loading N = 40	No Loading N = 20	*p*-Value *
Patients with bleeding complication, n (%)	10 (25)	11 (55)	0.212
*RBC transfusion, n patients* *Mean number of RBC transfusion*	10 (25) 2 [±6.1]	10 (50) 4.8 [±8.1]	0.053 0.139
*Intracranial bleeding, n*	0	1	0.429
ICU stay, mean	6.8 [±3.3]	12.2 [±9.8]	0.327
Hospital stay, mean	13.9 [±9.4]	14.9 [±12.7]	0.725
Survival to hospital discharge, %	77.5	60	0.156
Favorable neurological outcome at hospital discharge, % of survivors	94.1	82.4	0.318

Abbreviations: STEMI: ST-segment elevation myocardial infarction; RBC: red blood cell; ICU: intensive care unit. * Chi-square test/Fisher’s exact test in categorical variables and *t*-test in metric variables.

## Data Availability

The data presented in this study are available on request from the corresponding author. The data are not publicly available due to data protection of patients included.
